# Glioma Stem Cells as Immunotherapeutic Targets: Advancements and Challenges

**DOI:** 10.3389/fonc.2021.615704

**Published:** 2021-02-24

**Authors:** Keenan Piper, Lisa DePledge, Michael Karsy, Charles Cobbs

**Affiliations:** ^1^ Ben & Catherine Ivy Center for Advanced Brain Tumor Treatment, Swedish Neuroscience Institute, Seattle, WA, United States; ^2^ Sidney Kimmel Medical College, Philadelphia, PA, United States; ^3^ University of Washington School of Medicine, Spokane, WA, United States; ^4^ Department of Neurological Surgery, Thomas Jefferson University, Philadelphia, PA, United States

**Keywords:** glioblastoma stem cells, glioblastoma, cancer vaccination, radioresistance, tumor recurrence, cancer stem cell, brain tumors, immunotherapy

## Abstract

Glioblastoma is the most common and lethal primary brain malignancy. Despite major investments in research into glioblastoma biology and drug development, treatment remains limited and survival has not substantially improved beyond 1–2 years. Cancer stem cells (CSC) or glioma stem cells (GSC) refer to a population of tumor originating cells capable of self-renewal and differentiation. While controversial and challenging to study, evidence suggests that GCSs may result in glioblastoma tumor recurrence and resistance to treatment. Multiple treatment strategies have been suggested at targeting GCSs, including immunotherapy, posttranscriptional regulation, modulation of the tumor microenvironment, and epigenetic modulation. In this review, we discuss recent advances in glioblastoma treatment specifically focused on targeting of GCSs as well as their potential integration into current clinical pathways and trials.

## Introduction

Glioblastoma (GBM), a World Health Organization grade IV astrocytoma, is the most common primary brain malignancy with an incidence of 3.22:100,000 annually in the U.S (1). Despite standard of care treatment with maximal surgical resection, radiotherapy, adjuvant temozolomide (TMZ) chemotherapy, and tumor-treating-fields, median survival is still only 14.6 months (2), and nearly all patients succumb to fatal tumor recurrence and progression, with a <5% 5-year overall survival (OS).

The lack of improvement in GBM outcomes may be attributed, in part, to current therapies’ inability to target glioma stem cells (GSCs), a small subpopulation of cells that are implicated in tumor invasiveness, recurrence, and chemo(radio)resistance. The GSC population remains challenging both to define empirically and treat. GSCs are described by their ability to self-renew and differentiate to reform the heterogeneity of GBM (3). Multiple strategies to target GSC are currently under investigation with varying levels of preclinical and clinical development (4). In this review, we discuss the evidence supporting GBM’s common stem cell origin and outline the limitations of standard of care treatment for GBM. We then explore immunotherapeutic targeting of GSCs and highlight ongoing clinical trials.

## Glioblastoma and the Cancer Stem Cell Model

GBM development originally was defined by two divergent but interconnected models, namely, the stochastic model and CSC model. The stochastic or clonal evolution model suggests that all cells have the equal capacity for undergoing transformation based on accumulated mutations and/or epigenetic changes that confer a survival benefit (5). The CSC or hierarchical model suggests that a limited number of stem-like cells with few tumorigenic driver mutations have the capacity to divide symmetrically into identical daughter cells and differentiated progeny resulting in self-renewal and heterogeneous tumor progression (6–9). Bonnet and Dick’s seminal discovery of CSCs in leukemia, and subsequent discoveries of CSCs in most hematologic and solid tumor malignancies, including breast, colon, and some skin cancers greatly promoted the acceptance of the model (10–18). While CSCs mirror many of the features of normal stem cells, such as lineage determinization, resistance to apoptosis, neoangiogenesis, and self-renewal, they are distinct entities capable of tumorigenesis defined by few genetic mutations and altered epigenetic regulation (19–21). In the convergence of the stochastic and CSC models (22), differentiated cells can be transformed (23) and subsequently acquire a stem-like state (24), although some cell types such as neurons and their immediate precursors appear to be resistant to such mutations (25).

While the CSC hypothesis provides a compelling model for many cancers, attempts to define CSCs based on a set of genetic markers, epigenetic makeup, or cell state (e.g., quiescent or proliferative) have not reproducibly supported the isolation of fully competent CSCs. Three functional tests that are considered the gold standard for validating CSCs are: 1) self-renewal, 2) tumor initiation upon transplantation, and 3) differentiation into distinct progeny that can recapitulate the initial tumor’s heterogeneity upon serial transplantation (3). Regulation of the CSC population *in vitro* has depended on multiple molecular mechanisms, genetics, epigenetics, cellular states, intrinsic cell stimuli, microenvironmental influences, and other factors (7, 11–13). These mechanisms potentially allow the CSC population to transition between CSC and non-CSC states.

**Figure 1:**
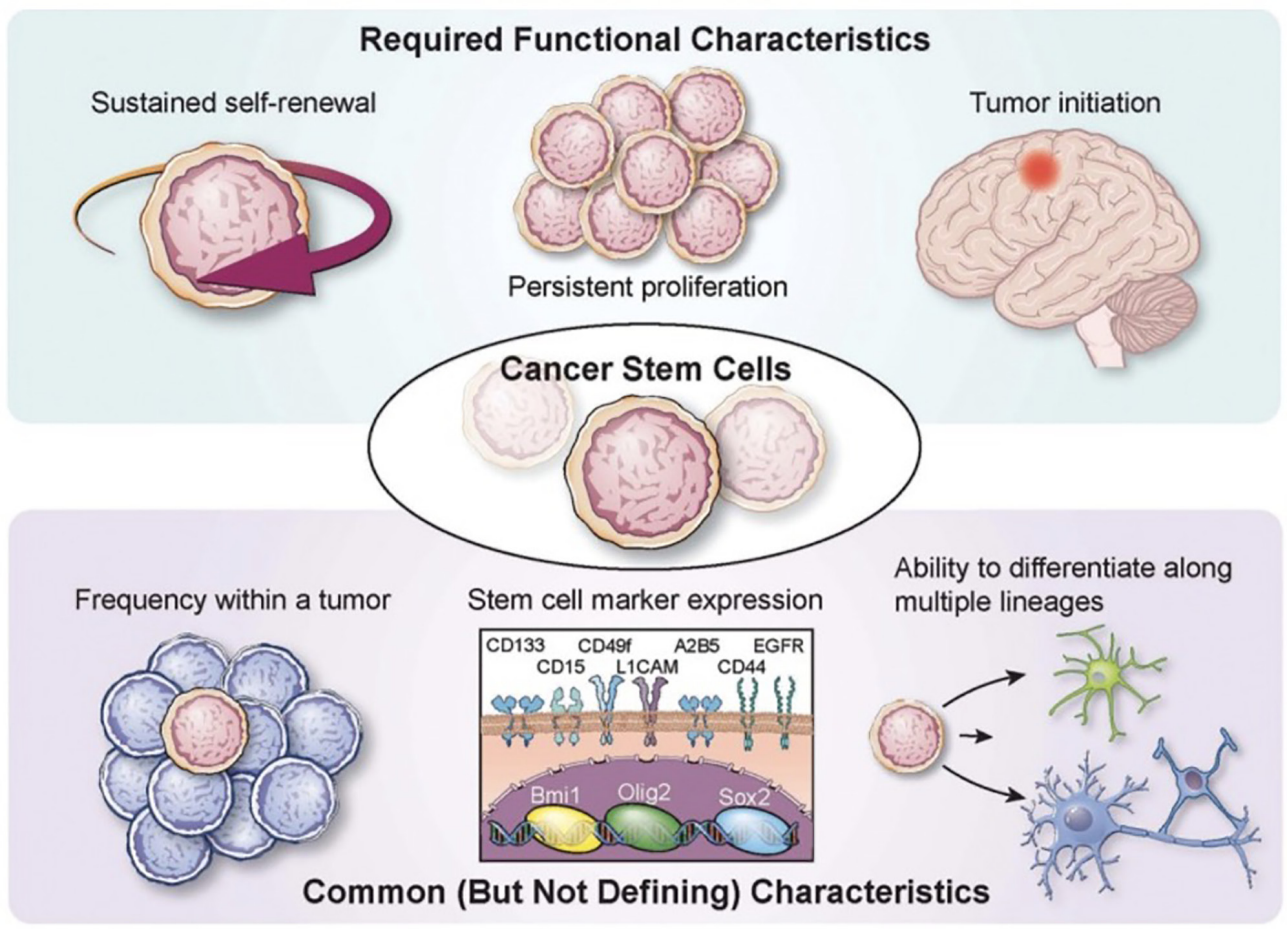
Characterization of glioma stem cells Various required functional characteristics of cancer stem cells (CSCs) and glioma stem cells (GCSs) are shown including self-renewal, proliferation, and initiation. Common characteristics include low frequency within a tumor, stem cell marker expression, and potential for differentiation. Reprinted with permission from Lathia et al. (3).

GSCs were first studied in 2002 (26), and were found to localize to a vascular niche (27). They are thought to arise from cells of the subventricular zone (SVZ) or differentiated glioma cells (28). Several markers of GSCs, namely, CD133, CD44, and CD15, help to define and enrich these population of cells but are not specific (3) (**Figure 1**). Recent single-cell sequencing studies have revealed that astrocyte-like neural stem cells with driver mutations migrate from the SVZ and lead to the development of high-grade gliomas in distant brain regions (28). This has provided precedent for radiotherapy targeted at high doses at the SVZ (National Clinical Trial [NCT] 02177578, NCT03956706). Another recent study demonstrated, *via* a xenotransplant model, the potential for slow-cycling cells to form rapidly cycling progenitor cells capable of self-maintenance and generation of non-proliferating progeny (29). These results are consistent with the CSC model suggesting that GBM tumor heterogeneity may result from a mono- or polyclonal tumor origin (30–32).

## Controversy

While there is evidence that supports GSCs involvement in GBM’s genesis, progression, and recurrence, there are several roadblocks to studying this cell population. First, stem cells are regulated in a multitude of ways, including genetic and epigenetic modifications, metabolic changes, cell responses to the immune system, microenvironment, and niche factors (3, 4, 33). These regulatory mechanisms result in a highly dynamic pool of cells which are therefore difficult to define and target. Additionally, the stem-like phenotype is mutable and in-vitro techniques may induce differentiation of the cells making them increasingly difficult to study. For the studies that have investigated, there are not consistent methods to define and isolate the physical characteristics of GSCs, so it is difficult to find consensus in the scientific community with regards to their role in GBM. Finally, CSCs in general are rare within the tumor mass (34), casting doubt upon the role they might play in tumor genesis, progression, and recurrence.

Despite the challenges of studying GSCs, there is hope that more advanced techniques, such as single cell sequencing, are elucidating some of the mysteries of these cells. One study by Patel et al. (2014) utilized single cell sequencing technology to investigate 430 cells in each of 5 GBM tumors, and elucidated a stem-like population of cells which existed within a stemness gradient (35). Further, a recent study by Couturier et al. (2020) used single cell RNA sequencing and discovered that in 16 IDHwt glioblastomas there was a GSC cell type with a distinct transcriptomic signature (36). While GSCs have historically been difficult to define, emerging technologies and findings are furthering the hypothesis that GSCs may be a worthy target in researching GBM therapeutics.

## Limitations of Current Glioblastoma Treatments

Current therapeutic treatment remains limited for GBM and multiple resistance mechanisms for GCS may partially account for this. Subclonal populations of cells left behind after gross total resection result in tumor recurrence and resistance (31). GSCs have the potential to maintain a quiescent cell cycle phenotype, rendering many chemotherapeutic agents ineffective. GSCs primarily reside within perivascular niches, where components of the extracellular matrix (ECM) modulate GSC survival and function. Several components within the ECM, like hyaluronic acid and the dystrophin–glycoprotein complex (DGC), have been shown to contribute to resistance and promote invasion (37–39). In addition, CSCs overexpress ATP-binding cassette (ABC) transporters that pump foreign toxins out of the cell, conferring multidrug resistance (40). Cells that express CD133, a cell surface marker highly associated with GSCs, have been shown to express O^6^-methylguanine-DNA methyltransferase (MGMT) at levels 32–56 times those of CD133(−) cells (41). The high activity of MGMT in these cells helps explain TMZ’s relative ineffectiveness against GSCs (42). By inducing expression of hypoxia-inducible factors, such as HIF1α and HIF2α—itself associated with poor prognosis for glioma patients (42)—TMZ may induce stemness in differentiated glioma cells (43). Resistance to radiation is also seen in GCSs, where a high ratio of GSCs to differentiated tumor cells correlates with increased tumor radioresistance (44), and CD133(+) cells have been reported to be resistant to apoptosis induced by *in vitro* radiotherapy (45). Hypoxic microenvironments preferentially contribute to GCS growth, which can reduce oxidative-stress produced by radiation (46). GSC radioresistance is also conferred by both the hypoxia-mediated activation of DNA damage checkpoint response enzymes Chk 1/2 (47) as well as by induction of autophagy to process and eliminate constituent parts of cells damaged by radiation (48).

## Targeted Therapy of GSCs

**Figure 2:**
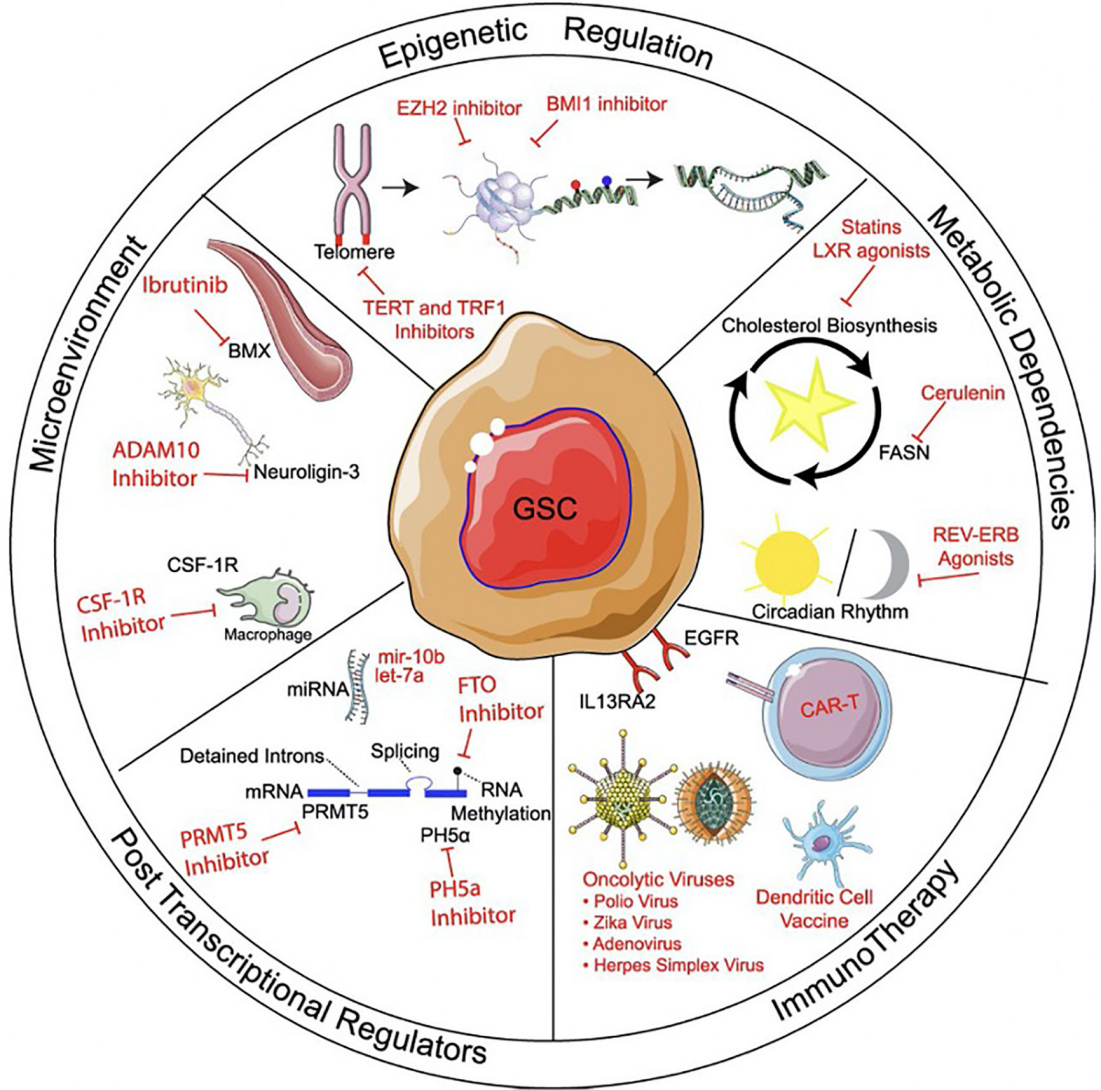
Methods of targeting glioma stem cells. Methods of targeting glioma stem cells (GSCs) can be divided into treatments targeting epigenetic regulation, metabolic pathways, microenvironment, post-transcriptional regulation, and immunotherapy. Within immunotherapy, strategies can include immunomodulatory drugs, oncolytic viral targeting, as well as passive, active, and adoptive immunotherapy approaches. Reprinted with permission from Gimple et al. (4).

Scientists have attempted to target GSCs through a multitude of avenues (**Figure 2**). Small molecules which activate or inhibit common, upregulated pathways in this cell population associated with their resilience. Targeted pathways are those that, when disrupted, result in chemo- and radiosensitization, tumor growth inhibition, induction of differentiation, inhibition of multidrug resistance, and promotion of apoptosis. Some of these pathways include STAT3 (49), Notch (50), PI3K/Akt/mTOR (51, 52), and Hedgehog (53). There are many pathways that contribute to the resilience and tumorigenicity of GSCs, and the dominant driver pathways vary from patient to patient. Because of this, finding a “one size fits all” small molecule solution is unlikely. Difficulties associated with chemotherapeutic approaches to treat GBM highlight the need for a new generation of cancer therapy, and immunotherapy presents an alternative that addresses these shortcomings.

## Immunotherapy: the Future of GSC Treatment?

The body prevents neoplastic proliferation primarily *via* the immune system. However, GBMs are known to exert immunosuppressive effects systemically and in the tumor microenvironment through a combination of decreased immunogenicity and active suppression of T cells that exceeds the immunosuppressive capacity of non-stem glioma cells (54, 55). Immunotherapy is a highly specific GBM treatment modality that may overcome the immunosuppressive effects of GBM generally, and GSCs in particular, through the introduction of monoclonal antibodies (mAb) or stimulation of the patient’s own immune response. These approaches may induce fewer side effects than other oncolytic methods which are less precise in their action.

**Figure 3:**
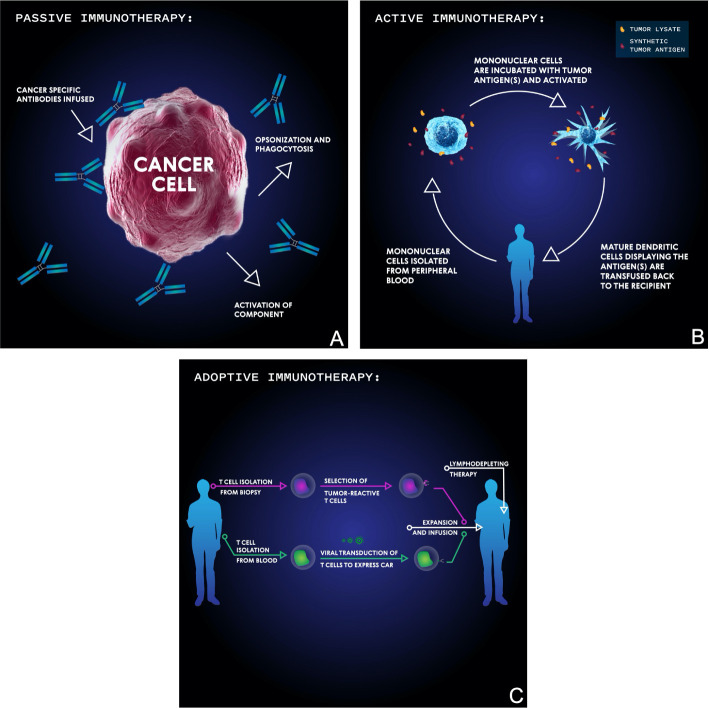
Basic schema of the main immunotherapeutic modalities for targeting malignancies. In passive immunotherapy **(A)**, antibodies are developed which bind specific tumor antigens and induce cellular-mediated phagocytosis or complement membrane attack complex-mediated cell death. In active immunotherapy **(B)**, mononuclear cells are isolated from the patient’s blood then incubated with synthetic or biopsy-derived tumor antigens and activated before being transfused back into the patient in order to facilitate an anti-tumor T cell immune response. In adoptive immunotherapy **(C)**, either tumor-infiltrating T cells are isolated from tumor biopsy, selected for their reactivity, and then transfused into a lymphodepleted patient, or T cells are isolated from blood, virally transduced to express a chimeric antigen receptor (CAR), and then transfused into a lymphodepleted patient.

Cancer immunotherapy approaches can be primarily categorized as passive, active, or adoptive (**Figure 3**). Passive immunotherapy uses antibodies to target tumor specific antigens and often doesn’t require a host immune response to initiate cancer cell death. Active immunotherapy activates the host’s immune system against tumor specific antigens, most often utilizing dendritic cells as antigen presenters. In adoptive immunotherapy, immune cells are removed from the patient, selected or genetically engineered for their reactivity against a target of interest, and reintroduced. Finally, many consider virotherapy a form of immunotherapy due to the activation of the immune system. Virotherapy makes use of genetically engineered oncolytic viruses to train the body’s immune system against remnant cancer particles following virus-mediated killing.

Radiation therapy used as a complement with immunotherapy is an exciting and promising avenue to explore in the management of GBM. For example, radiation has been shown to cause immunogenic tumor cell death (ICD). ICD induces the translocation of calreticulin to the surface of the tumor cells surface, causing APCs to phagocytose tumor cells. ICD also promotes the release of HMGB1, encouraging dendritic cell maturation and tumor antigen presentation (56). Radiation can also enhance the permeability of the blood-brain barrier, which may allow T-cells to invade the tumor. Furthermore, radiation has been shown to drastically increase the presence of MHC on the tumor cell surface providing greater density of T-cell targets (57–60). The various potential benefits presented by the combination of radiation and immunotherapy have spurred immense interest in the field.

In this section, we will discuss immunotherapy clinical trials which target GSC-specific and GSC-overexpressed targets with a focus on ongoing clinical trials (**Table 1**).

**Table 1:** Ongoing immunotherapy clinical trials targeting glioma stem cells (GSCs).

Trial name	Therapy type	Target	Combination	Phase (O-III)	ClinicalTrials.gov Identifier
AVeRT	DC vaccine	pp65	Nivolumab	I	NCT02529072
DENDR-STEM	DC vaccine	Autologous GSCs		I	NCT02820584
IL13R⍺2 CAR T cell therapy	CAR T cell	IL13R⍺2	Ipilimumab, nivolumab	I	NCT04003649
Allogeneic GSC lysate DC vaccine	DC vaccine	Allogeneic GSC lysate	SOC	I	NCT02010606
HERT-GBM	CAR T cell	HER2, pp65		I	NCT01109095
Autologous GSC lysate DC vaccine	DC vaccine	Autologous GSC lysate	SOC	II	NCT01567202
SurVaxM	Antibody mediated T cell therapy	Survivin	TMZ, GM-CSF	II	NCT02455557
ELEVATE	DC vaccine	pp65	Td, basiliximab, TMZ	II	NCT02366728
ATTAC-II	DC vaccine	pp65	TMZ	II	NCT02465268
AV-GBM-1	DC vaccine	Autologous tumor-initiating cellular antigens		II	NCT03400917
DEN-STEM	DC vaccine	GSC antigens, hTERT, survivin		II/III	NCT03548571

GM-CSF, granulocyte-macrophage colony-stimulating factor; GSC, glioma stem cell; SOC, standard of care; TMZ, temozolomide; Td, tetrodotoxin.

### Passive Immunotherapy

Passive immunotherapy utilizes antibodies to bind to oncomodulatory signaling molecules or target proteins on cancer cells and disrupt cellular function without producing a memory immune response in the patient. The most significant improvement in standard of care treatment in the United States recently has been the addition of the monoclonal antibody bevacizumab, or Avastin, which was granted accelerated approval by the FDA in 2009 (61). Bevacizumab targets vascular endothelial growth factor (VEGF), a signaling molecule that promotes angiogenesis and is secreted in high quantities by GBM cells. While bevacizumab has improved the progression free survival (PFS) of patients with GBM, it has failed to improve OS. GSCs are notoriously resistant to hypoxia and may therefore persist despite the additional therapeutic, contributing to inevitable recurrence (62).

Epidermal growth factor receptor (EGFR) is molecular target overexpressed in GSCs which confers chemo- and radioresistance in GBM tumors, resulting in poorer outcomes among GBM patients (63). The EGFR-targeting mAbs nimotuzumab and cetuximab have been shown to reduce the total number of radioresistant CD133(+) cancer stem cells in a murine glioma model (64). Nimotuzumab alone demonstrates antiangiogenic and antiproliferative activity while cetuximab inhibits downstream EGFR signaling, resulting in tumor radiosensitization. The co-administration of these drugs delayed tumor growth, decreased brain tumor sizes, inhibited invasion, and promoted tumor cell apoptosis. The synergistic effects of these monoclonal antibodies makes the case for further investigation of combination therapies, especially given the well-documented resistance developed by GBM tumors to individual anti-EGFR mAbs which are frequently rendered ineffective by extracellular EGFR mutations (65, 66). This limitation has been evidenced in clinical trials when, used in combination with standard of care treatment, nimotuzumab failed to demonstrate significantly improved PFS or OS in 142 patients with newly diagnosed GBM (NCT00753246) (66). Cetuximab is now being assessed in a phase I/II clinical trial in combination with bevacizumab (NCT01884740). There are currently nearly two dozen trials testing the efficacy of bevacizumab in combination with another treatment against GBM (67).

EGFR variant III (EGFRvIII) is a constitutively active mutated form of EGFR that is highly expressed in many GBM tumors (68). Though not specific to GSCs, it is significantly co-expressed with CD133 (69) and promotes a stem-like phenotype in GBM cells. It has been targeted for antibody therapy in combination with radiation and chemotherapy. Although the anti-EGFRvIII antibody rindopepimut showed promising results in phases I and II (70, 71), its large international phase III trial, ACT IV, was discontinued after interim analysis did not demonstrate survival benefit (72).

While monotherapies have stumbled in clinical studies, bispecific antibodies (bsAb) and novel technologies have shown promise in bolstering the anti-GSC effects of passive immunotherapies. A bispecific antibody against CD133 and EGFRvIII was demonstrated to be highly cytotoxic against GSCs (but not NSCs) and significantly more effective in prolonging OS in mice as compared to CD133 or EGFRvIII mAbs alone (69). Though it has yet to be validated in human studies, these bispecific antibodies’ increased specificity may confer greater anti-GSC effects and decreased toxicity than monotherapies (73, 74). Near-infrared photoimmunotherapy (NIR-PIT) is another novel technology that has the potential to improve the anti-GSC effect of monoclonal antibodies. NIR-PIT involves administration of monoclonal antibodies tagged with photoactive molecules (commonly IR700 dye) followed by near-infrared irradiation. Photoactivation of these antibodies results in specific and robust cell death *via* cellular membrane damage. Jing et al. demonstrated that CD133-targeted NIR-PIT induced rapid cell death of CD133(+) GSCs *in vitro* and in orthotopic GSC tumor mouse models (75). Importantly, the ability to administer this non-harmful irradiation through the skull suggests that NIR-PIT may present a safe treatment method in humans.

While research into GSC-specific passive immunotherapy is sorely lacking, additional research is warranted given their demonstrated superiority to bulk tumor-targeting mAbs in preclinical applications. New antibodies which can eliminate chemo(radio)resistant GSC populations, such as one against anti-apoptotic protein CD47, are being developed constantly and warrant optimism (74). Given their ability to target a variety of pathways, and their general tolerability in humans, GSC-specific passive immunotherapies may be utilized as safe and effective adjuncts to more aggressive chemo- or immunotherapeutic approaches.

### Active Immunotherapy

Both dendritic cell (DC) vaccines and antibody-mediated T cell immunotherapies rely on the activation of host immunity in order to target specific cancer cells. These approaches have demonstrated safety and efficacy for treatment of GBM in both preclinical and clinical trials (76). Given the success of these trials, researchers are utilizing active immunotherapeutic approaches to eliminate chemo(radio)resistant GSC subpopulations. These GSC specific therapies work *via* two primary mechanisms: 1) promotion of a broad immunity against GSCs by GSC lysate-pulsed DCs and 2) activation of immunity against specific GSC antigens by synthetic peptide/RNA/mRNA-pulsed DCs.

Promoting immunity against GSC lysate trains the immune system against any antigens associated with GSCs. Murine models which utilize this technique have highlighted the potential for anti-GSC DC vaccines and have served as the basis for multiple clinical trials. Dendritic cells pulsed with GSC tumor lysate have shown to be highly effective at preventing viability of murine GBM tumors grown as both neurospheres (which preferences GSC growth) (77), and have elicited specific T cell responses against GSCs and improved OS in xenografted mice (78). Allogenic GSC lysate-loaded DCs are now being tested in multiple clinical trials (NCT02010606 and NCT01567202).

Other groups are utilizing patients’ surgical specimens to culture tumor-initiating GSCs and train autologously derived dendritic cells (NCT03400917). Along with GSC lysate, it is also possible to extract mRNA from patient derived GSCs and produce personalized vaccines. One phase I trial, NCT00846456, demonstrated the safety of this approach as well as a nearly three-times longer PFS compared to matched controls (78).

Much effort has been taken over the past three decades to define GSC specific peptides in order to decrease the risk for off-target effects, and numerous clinical studies are now assessing the effectiveness of DC vaccines which target peptides that are highly expressed in GSCs. One phase III clinical trial, NCT02546102, which was suspended in 2017 for inadequate funding showed significant promise in early phases. DC vaccines were pulsed with 6 synthetic peptides overexpressed in GSCs: HER2, TRP-2, gp100, MAGE-1, IL13R⍺2, and AIM-2 and this therapy was given in conjunction with standard of care chemo(radio)therapy. Results from phase I of the trial suggested a powerful therapeutic effect: median PFS in newly diagnosed patients treated with the vaccine was 16.9 months and OS was 38.4 months, noticeably exceeding historical standards (79). Five patients who underwent a second tumor resection also demonstrated a decrease or absence of CD133 expression in tumor tissue, suggesting the therapy may have exerted GSC-selective cell death. Another phase I trial (NCT02049489) demonstrated that a DC vaccine against CD133 was well tolerated in a pilot group of 20 patients (80).

The phosphoprotein pp65, a product of human cytomegalovirus (HCMV), is an interesting DC vaccine target. A large fraction of clinically isolated CD133(+) cells are found to be positive for pp65, and infection of GBM cells with HCMV *in vitro* causes an upregulation of CD133, Notch1, Sox2, Oct4, and Nestin, and promotes the growth of GBM neurospheres, suggesting that pp65 may play a role in stemness (81). The presence of HCMV in GBM is a controversial topic, having been confirmed and denied by various labs (82). Regardless, targeting HCMV products may show promise in clinical trials.

Researchers at Duke University have a number of ongoing clinical trials investigating the effectiveness of a pp65 RNA-pulsed DC vaccine in combination with various other treatments. One addition being tested is that of anti-IL-2R⍺ antibodies which researchers hope will decrease Treg function and improve the penetration of DC vaccinations. Early research demonstrated that TMZ-induced lymphopenia enhances vaccine responses but dramatically upregulates T-reg function. IL-2R⍺ therefore diminishes the T-reg response allowing for a more robust anti-tumor effect (83). Interestingly, administration of IL-2R⍺ antibodies depleted vaccine-induced immune responses in mice without lymphopenia, but acted synergistically with TMZ in mice experiencing TMZ-induced lymphopenia. These results were confirmed in a pilot study with six patients and draw focus to the importance of combination therapies which utilize tumor debulking therapies like TMZ along with targeted therapies like IL-2R⍺ antibodies and DC vaccines.

In a phase I trial (NCT00626483), the group combined a pp65 DC vaccine with basiliximab, another anti-IL-2R⍺ antibody (84). Initial results from phase I of the trial demonstrated high tolerability of the combination therapy but survival benefit was not extrapolated at the time of data collection. After demonstrating that pp65 RNA-pulsed DC vaccines with tetanus/diphtheria (Td) toxoid pre-conditioning significantly increased patient PFS and OS in a small pilot study (85), the group is comparing the effectiveness of pp65 vaccine alone, with Td toxoid pre-conditioning, and with both Td toxoid pre-conditioning and basiliximab against newly diagnosed GBM after standard of care treatment (ELEVATE; phase II; NCT02366728). The most promising of the group’s trials combines the pp65 DC vaccine with dose-intensified TMZ cycles (ATTAC; NCT00639639). Preliminary results demonstrated more than double the OS and triple the PFS as compared to historical controls (85). Notably, four patients remained progression-free at 59 to 64 months. Phase II of this trial (NCT02465268) is currently underway. Finally, an ongoing phase I trial (NCT02529072) is evaluating the effectiveness of the pp65 vaccine in combination with the programmed cell death 1 (PD-1) blocking antibody nivolumab. In a fashion similar to anti-IL-2R antibodies, the inclusion of this checkpoint inhibitor will hopefully antagonize GBM’s immunosuppressive effects.

Another promising ongoing clinical trial is that of a trivalent GSC-targeting DC vaccine which is now in stage II/III (NCT03548571). Researchers at Oslo University Hospital are targeting GSCs by administering DC vaccines transfected with GSC mRNA along with the anti-apoptotic peptide survivin and human telomerase reverse transcriptase (hTERT). Both survivin and hTERT have been found to increase stemness in GBM and are expressed in high levels in GSCs (86, 87). In a small preliminary study of this therapy, median PFS was nearly three times longer as compared to those receiving standard of care.

Monoclonal and bispecific antibodies are also being investigated for their ability to activate T cell immune responses against GSCs, and once again survivin is a promising target. SurVaxM, a survivin vaccine, is being tested in combination with TMZ and granulocyte-macrophage colony-stimulating factor in a phase II clinical trial (NCT02455557). Initial results warrant optimism: of 55 patients with newly diagnosed GBM treated with the SurVaxM vaccine concomitant with standard of care therapy, 96% were progression free at 6 months and 93% were alive at 12 months, a substantial improvement over historical controls of 43 and 41%, respectively (88). Survivin has also been utilized along with peptides IL13R⍺2 and Ephrin-A2, a target highly expressed in GSCs and responsible for self-renewal and tumorigenicity (89). This trivalent vaccine was tested in a phase I/II trial (NCT02078648) against recurrent GBM with or without bevacizumab but demonstrated poor results, with median OS around 11 months in both groups (90).

The recombinant bispecific antibody AC133CD3 targets T cells and the CD133 epitope AC133, redirecting human polyclonal T cells to patient derived AC133(+) GSCs, inducing GSC lysis, and preventing the growth of subcutaneous GBM xenografts (91). In tandem with CD8(+) T cell infusion, this treatment has been demonstrated effective as both a prophylactic and therapeutic treatment for orthotopic GSC-derived brain tumors, while AC133(+) hematopoietic stem cells were virtually unaffected by the therapy.

The successes of endogenous and virus-associated GSC antigen-targeted therapies indicate that GSC antigens may represent promising targets for various therapy modalities. Caution must be taken, however, when considering early clinical victories, particularly with antibody-mediated T cell therapies. Some trials which demonstrated promise in phase I and II have failed in phase III as they could not demonstrate survival benefit. In the context of their established success in recurrent GBM treatment, DC vaccines are a promising GBM immunotherapy approach and preclinical and clinical results of GSC antigen-specific and GSC lysate DC vaccine approaches should motivate further investigation.

### Adoptive Immunotherapy

Adoptive immunotherapy utilizes a patient’s own immune cells—whether selected or genetically modified for antitumor activity—to combat the growth and spread of neoplasia. Both T cell and natural killer (NK) cell therapies are of growing interest for various cancers and are being utilized to target GSCs. Cytotoxic T lymphocytes (CTL) can be used to target tumor-associated antigens by being drawn from the patient, selected for their existing antitumor specificity, and expanded *ex vivo* before autologous reintroduction. CTL-mediated GSC targeting has demonstrated promise in preclinical applications and small human cohorts, but has yet to be put to test in a large clinical trial. Chimeric antigen receptor (CAR) T cells, in contrast, are genetically engineered to exert their cytotoxic effects against specific antigens and have been extensively studied in the context of targeting tumor-associated antigens in various types of cancer. Though CAR T cell therapies have been successful treating blood cancers—two different therapies were approved by the FDA in 2017 for treatment of acute lymphoblastic leukemia (92) and diffuse large B cell lymphoma (93)—they have shown mixed results in targeting solid cancers. NK cell therapies are less common and represent a promising, but largely theoretical, avenue for targeting GSCs. NK cells broadly recognize transformed cells, do not require activation by particular tumor-bound antigens, and generally leave healthy cells unharmed. Finally, CAR NK cells, like CAR T cells, are genetically modified NK cells which target cancer-specific antigens. Researchers hope to harness these technologies to eliminate chemo(radio)resistant GSC populations while relying on standard of care therapy to debulk tumors.

CAR T cells have been developed to target several GSC-specific antigens. In a preclinical application of CAR T cell therapies targeting GSCs, therapies have been developed against the CD133 epitope AC133 (94). These AC133-specific CAR T cells recognized and eradicated patient-derived AC133(+) glioma stem cells *in vitro* and in mouse models and improved OS in treated mice.

Non-GSC-specific peptides, which are also upregulated in GSCs, have demonstrated some efficacy in killing GSC populations. IL13Rα2 (95, 96), EGFRvIII (97, 98), and chlorotoxin-based therapies (99) have all been shown to eliminate both GSCs and bulk tumor cells in preclinical experiments. An IL13Rα2 CAR T cell therapy is currently being investigated in a phase I clinical trial alone and in combination with two checkpoint inhibitors (NCT04003649).

Adoptive immunotherapies cannot always be easily subcategorized. Some groups of researchers are investigating technologies which utilize both genetic modification (i.e., CAR) as well as selection of T cells based on reactivity to particular antigens (i.e., CTL). A group at Baylor has an ongoing clinical trial (NCT01109095) aiming to improve the efficacy of a human epidermal growth factor receptor 2 (HER2) CAR T cell therapy by selecting for cytotoxic T cells which recognize the human cytomegalovirus (HCMV) protein pp65. The group has previously demonstrated the efficacy of the HER2-targeted CAR T cells in eliminating GBM cells irrespective of CD133 expression (100). Their inclusion of the pp65 target in this clinical trial is predicated on the theory that anti-HCMV antibodies are present in most human adults and thus this HCMV protein will cause persistent activation of the CAR T cells. In addition to broadly activating the CAR T cells, it is possible that this treatment preferentially targets GSCs. As previously mentioned, HCMV has been shown to increase stemness of GBM cells, and pp65 is preferentially expressed in GSCs among infected tumors (81). Initial results from the phase I study demonstrate that the approach is safe and potentially effective (101). Eight out of the 16 patients enrolled demonstrated objective response to the treatment. Three of the patients demonstrated stable disease and were still alive 24 to 30 months, and longer, after T cell infusion.

Some groups also are investigating the HCMV protein pp65 as a target for CTL-mediated oncolysis. In one study, HCMV pp65-specific CTLs were comparably cytotoxic against both GSCs and differentiated cells both *in vitro* and in a mouse model (102). Here, all GSC populations were eliminated *in vivo* in an antigen-specific manner, indicating a potentially safe method of attacking GBM. Two phase I/II clinical trials (NCT01205334 and NCT00990496) which utilized pp65-trained CTLs in patients with GBM were stopped due to poor subject recruitment, though other groups are pursuing this approach with promising preliminary results. The Duke group pursuing multiple pp65 DC vaccination trials has also demonstrated that training T cells with pp65-pulsed DCs increases the polyfunctionality of CTLs in a cohort of 11 patients (NCT00693095), increasing OS (103). Another group at MD Anderson is investigating autologous pp65-specific CTLs following lymphodepleting doses of TMZ and found that the therapy was well tolerated in a pilot trial of 12 patients (104). Unfortunately, the group recently released results of their phase I/II trial which demonstrated attenuated T cell functionality and poor PFS (NCT02661282) (105).

Another peptide which is being utilized for CTL-mediated cytolysis is that of SOX6, an immunogenic peptide is involved in inhibition of neuronal cell differentiation and neuronal stem cell maintenance (106, 107). It has been demonstrated that, due to SOX6’s immunogenicity and upregulation in GSCs, human leukocyte antigen (HLA)-A2, and -A24 restricted SOX6 derivatives are effective and safe targets for glioma CTL-mediated cytolysis in mouse model (107). This target has yet to be tested in a clinical setting but represents another avenue for CTL therapies targeting GSCs.

Most adoptive GBM immunotherapy research has been devoted to CAR T cell and CTL therapies as early research suggested NK cells were ineffective against GBM (108–110). However, in 2009, Castriconi et al. showed that allogeneic and autologous IL-2 and IL-5-activated NK cells were effective in killing human-derived GSCs (111), opening the door to further investigations into lymphokine-activated NK cell therapy. Recent research supports this finding and suggests that NK cell therapies might be even more effective against GSCs than differentiated cells, as GSCs were significantly more susceptible to NK cell-mediated cytolysis than were cells grown in differentiation-inducing media (112).

Finally, CAR-NK cells have also been explored for their ability to selectively target and eradicate GSCs. In 2015, a group from The Ohio State University demonstrated that CAR-NK cells targeting both EGFR and EGFRvIII effectively killed patient-derived GSCs *in vitro* (113). They also demonstrated that EGFR targeting CAR-NK cells significantly suppressed growth of xenografted human-GSC tumors in mice and yielded double the median lifespan compared to controls. Of note, the EGFR targeting CAR-NK cells were more effective than non-modified NK cells.

Preclinical insights for NK and CTL therapies warrant some optimism, and clinical evidence that utilizing DCs to train CTLs increases the treatment modality’s effectiveness supports further research into this strategy. However, in the absence of clinical evidence which demonstrates an improvement in OS or PFS, hope for approaching advancements in adoptive GSC therapies lies primarily with CAR T cells. Even these advancements, though, will take patience. None of the three ongoing CAR T cell clinical trials which target GSCs have yet to reach phase III, and therefore clinical adoption is unlikely for a number of years.

### Virus-Mediated Immunotherapy

A broad range of viruses have been explored for treating high grade glioma (114), and the list continues to grow (115). These viruses have demonstrated tropism for tumor cells resulting in tumor cell lysis, recruitment of the immune system, and finally a T cell mediated antiviral and antitumor response. This leads to systemic immunity against the tumor and its recurrence (116). Because of the interaction with the immune system, some have classified the use of oncolytic viral therapies as an immunotherapeutic approach. Clinical trials involving viral immunotherapies against GBM have recently been reviewed (117), and those which target GSCs in addition to bulk tumor cells are listed in **Table 2**. Researchers have focused efforts on fine tuning genetic modifications in viruses to specifically target tumor cells and introduce cytotoxic transgenes. Transgenes generally function by enhancing prodrug activation, inducing apoptosis and immune activation, and in the case of GBM, inhibiting angiogenesis. Investigators have utilized these features to deliver short interfering (si)RNA and short hairpin (sh)RNA in order to downregulate cancer cell gene expression, or directly kill GSCs. Lentivirus-mediated shRNA inhibition of Chk1/2, the stem cell gene SirT1, and STAT3 have all been found to sensitize GSCs to radiotherapy (45, 47, 118, 119). Clinical trials have had varying success, and like most therapies for GBM, clinical adaptation is slow. To date, there are only two governmentally approved oncolytic virus-mediated immunotherapies on the market, both of which utilize herpes simplex virus vectors (120, 121).

**Table 2:** Ongoing virotherapy clinical trials targeting glioma stem cells (GSCs).

Virus family	Therapy type	Viral strain	Combination	Phase (O-III)	ClinicalTrials.gov Identifier
ADV	NSC vector oncolytic virus	BM-hMSC-DNX-2401		I	NCT03896568
Reovirus	Oncolytic virus	Wild-type reovirus (reolysin)	Sargamostim (rGM-CSF)	I	NCT02444546
Vaccinia virus	Oncolytic virus	TG6002	5-FC	I	NCT03294486
ADV	Oncolytic virus	DNX-2440		I	NCT03714334
HSV	Oncolytic virus	HSV G207	Low dose radiation	I	NCT03911388
HSV	Oncolytic virus	C134-HSV-1		I	NCT03657576
ADV	Viral vector gene therapy	ADV/HSK-tk	Valacyclovir, SOC	I	NCT03596086
ADV	Viral vector gene therapy	ADV/HSK-tk	Valacyclovir, SOC	I	NCT03603405
ADV	Oncolytic virus	DNX-2401	Pembrolizumab	II	NCT02798406

ADV, adenovirus; HSV, herpes simplex virus; rGM-CSF, recombinant granulocyte-macrophage colony-stimulating factor; 5-FC, flucytosine; SOC, standard of care.

## Discussion

The growing body of knowledge regarding GSCs’ role in oncogenesis and tumor recurrence necessitates reevaluation of conventional cancer assessment and treatment methods, as lasting remission will likely remain elusive for many GBM patients without the means of reliably detecting and extinguishing GSC populations. Methods of targeting GSCs vary widely, both in terms of the vector used to exert cytolytic effects on this cell population as well as the antigen or cellular pathway targeted by such vectors. Over the past several years, immunotherapy has emerged as a promising method of culling GSC populations in GBM tumors and has increased survival in GBM patients. Nevertheless, immunotherapies targeting malignancies of the immune-privileged central nervous system present a host of unique challenges.

Due to the relative inability of many immunotherapies to penetrate the blood-brain barrier and subsequently localize to GBM, modalities which show promise *in vitro* may stumble in *in vivo* and clinical application. Direct introduction of immunotherapeutic agents into the tumor resection cavity effectively bypasses the blood-brain barrier but runs the risk of causing inflammation in the brain due to a productive immune response. The paradoxical need for a robust immune response with limited inflammatory changes to kill GBM while preserving the patient highlights just one of the complexities of immunotherapy in the context of intracranial neoplasia. Dexamethasone, the current standard of care agent to treat GBM-associated edema, exerts global immunosuppression and may thereby limit the efficacy of some immunotherapies (122). This underscores the importance of developing safe anti-inflammatory medications to be administered concomitantly with immunotherapy that do not limit the effectiveness of these agents.

Another hurdle for future immunotherapies to clear is the necessity that therapies be highly specific for glioma stem cells, avoiding antigens shared by healthy neural stem cells or other normal stem cell populations throughout the body, in order to mitigate the risk of adversely affecting somatic stem cell function. Even with highly targeted therapies, however, GBM tumors’ innate immunosuppressive effects can dampen the benefit of immunotherapies. Although aberrant cell growth results in immune recruitment through production of chemotactic factors, GBMs antagonize this process by secreting other chemokines which recruit T regulatory cells and suppress immune effector cells (123). In GBM, systemic decrease in T cell responsiveness and immunoglobulin levels, as well as an increase in Treg circulation, limits the effectiveness of those immunotherapies which rely on the body’s endogenous immune system. Further, immunosuppression in the tumor microenvironment can render ineffective both endogenous and exogenous immunotherapies. Finally, autologous vaccination strategies are both costly and time consuming. Given the rapid progression of GBM and the association between minimal tumor burden and immunotherapy success (124), efforts to expedite vaccine preparation are imperative to the success of this treatment modality.

Though many immunotherapeutic approaches are currently being investigated, some have shown greater promise in clinical application than others. The use of static monotherapies such as single-target antibodies have been repeatedly demonstrated to be ineffective in the long term due to cellular adaptation by GSCs and, in some cases, even differentiated tumor cells. However, the success of bevacizumab in slowing GBM progression and improving quality of life indicates that passive immunotherapies might be viable adjuncts to more aggressive chemo- or immunotherapeutic approaches.

Further, research into the interaction between GSCs and the tumor microenvironment has shown us that stem cell phenotypes vary significantly throughout the tumor and that subpopulations of GSCs may be differentially susceptible to immunotherapeutic approaches (125). These studies provide a basis for pursuing multiple immunotherapeutic modalities based on the relative permissiveness of GSC populations. Currently, the most thoroughly investigated and perhaps most promising form of immunotherapy against GBM is that of DC vaccination. Nearly three dozen clinical trials are currently assessing DC vaccines against high grade gliomas (126). DC vaccines’ ability to train cytotoxic T cells to target GSCs without harmful off-target effects is well documented in both preclinical and clinical applications. However, none of these vaccines have yet attained FDA approval. The number of avenues being pursued to target GSCs warrants optimism, but for now agents trained to eliminate this cancer-driving cell population remain inaccessible to many.

## Author Contributions

KP, LD, MK, and CC all contributed to the writing and editing of the manuscript. All authors contributed to the article and approved the submitted version.

## Funding

This work was supported by the Ben & Catherine Ivy Center for Advanced Brain Tumor Treatment General Fund.

## Conflict of Interest

The authors declare that the research was conducted in the absence of any commercial or financial relationships that could be construed as a potential conflict of interest.
